# Kinetics of Anti-*Phlebotomus perniciosus* Saliva Antibodies in Experimentally Bitten Mice and Rabbits

**DOI:** 10.1371/journal.pone.0140722

**Published:** 2015-11-16

**Authors:** Inés Martín-Martín, Ricardo Molina, Maribel Jiménez

**Affiliations:** Unidad de Entomología Médica, Servicio de Parasitología, Centro Nacional de Microbiología, Instituto de Salud Carlos III, Majadahonda, Madrid, Spain; Pasteur Institute of Iran, ISLAMIC REPUBLIC OF IRAN

## Abstract

**Background:**

Sand flies are hematophagous arthropods that act as vectors of *Leishmania* parasites. When hosts are bitten they develop cellular and humoral responses against sand fly saliva. A positive correlation has been observed between the number of bites and antibody levels indicating that anti-saliva antibody response can be used as marker of exposure to sand flies. Little is known about kinetics of antibodies against *Phlebotomus perniciosus* salivary gland homogenate (SGH) or recombinant salivary proteins (rSP). This work focused on the study of anti-*P*. *perniciosus* saliva antibodies in sera of mice and rabbits that were experimentally exposed to the bites of uninfected sand flies.

**Methodology/Principal Findings:**

Anti-saliva antibodies were evaluated by ELISA and Western blot. In addition, antibody levels against two *P*. *perniciosus* rSP, apyrase rSP01B and D7 related protein rSP04 were determined in mice sera. Anti-saliva antibody levels increased along the immunizations and correlated with the number of sand fly bites. Anti-SGH antibody levels were detected in sera of mice five weeks after exposure, and persisted for at least three months. Anti-apyrase rSP01B antibodies followed similar kinetic responses than anti-SGH antibodies while rSP04 showed a delayed response and exhibited a greater variability among sera of immunized mice. In rabbits, anti-saliva antibodies appeared after the second week of exposure and IgG antibodies persisted at high levels, even 7 months post-exposure.

**Conclusions/Significance:**

Our results contributed to increase the knowledge on the type of immune response *P*. *perniciosus* saliva and individual proteins elicited highlighting the use of rSP01B as an epidemiological marker of exposure. Anti-saliva kinetics in sera of experimentally bitten rabbits were studied for the first time. Results with rabbit model provided useful information for a better understanding of the anti-saliva antibody levels found in wild leporids in the human leishmaniasis focus in the Madrid region, Spain.

## Introduction

Leishmaniasis is a parasitic disease, where the protozoan -*Leishmania* spp.- is the causative agent while several species of phlebotomine sand flies (Diptera: Psychodidae) serve as vectors [1]. Sand fly saliva contains a complex cocktail of anti-hemostatic and immunomodulatory molecules that are inoculated into host skin during blood-feeding. Many of the salivary proteins are immunogenic and promote the development of specific cellular and humoral immune responses [2]. Concretely, cellular immunity is known to play a role in the protection against the establishment of *Leishmania* parasite due to pre-exposure to sand fly bites [3]. Although anti-saliva antibody response is not responsible for the leishmaniasis protection, detection of anti-saliva antibodies can be used as a marker of exposure to sand flies as the appearance of antibodies against these arthropod salivary proteins is specifically dependent on the exposure. The correlation between IgG anti-saliva antibody levels and exposure to blood-sucking arthropods was evidenced for the first time in sera of farmers who had been exposed to *Ixodes damini* bites in New Jersey [4]. Subsequently, additional serological studies have shown that anti-saliva antibody kinetics are seasonal and coincident with the period of activity of arthropods [4–6].

Measuring anti-saliva antibodies as a marker of exposure can be a useful tool in epidemiological studies as a simple way to determine the effectiveness of vector control campaigns. The reduction of specific anti-saliva antibody levels in sera of hosts after the incorporation of vector control measures imply a success in the campaign, just as it has been demonstrated in Angola or Nepal following the use of impregnated mosquito nets in endemic areas of malaria and leishmaniasis respectively [7, 8]. As screening of specific anti-saliva antibodies is limited by the availability of salivary gland homogenate and the protein variability found among species and specimens, utilization of recombinant salivary proteins has become a promising alternative. However, little is known about the kinetics of the humoral response elicited by specific salivary proteins.

To date, only a few studies have focused on the analysis of kinetics of anti-sand fly saliva antibodies in mice, dogs and humans [9–15]. To our knowledge, no data regarding kinetics of specific anti-sand fly antibodies in leporids are available. Hares and rabbits (*Lepus granatensis* and *Oryctolagus cuniculus* respectively) have been recently demonstrated to act as *Leishmania infantum* reservoirs in a human leishmaniasis outbreak in the southwestern area of Madrid, Spain [16, 17]. The large number of human cases (616 reports from 2010 to February 2015) has led to an increased incidence from 2.44/100,000 inhabitants in 2009 to 49.0/100,000 inhabitants in 2014 in Fuenlabrada, the most affected municipality (Community of Madrid, personal communication) and it is considered the largest focus of visceral leishmaniasis described in Europe.

Therefore, the aim of this study was to expand the knowledge on anti-*Phlebotomus perniciosus* salivary antibody kinetics using a BALB/c mice and a rabbit model. In addition, kinetics of antibodies against two recombinant salivary proteins (rSP): rSP01B and rSP04 were conducted.

## Methods

### 2.1. Sand flies and salivary glands collection

*P*. *perniciosus* sand flies were maintained at 27°C and 17:7 light-darkness photo-period at the Medical Entomology Unit of the Instituto de Salud Carlos III (ISCIII), Madrid, Spain. Sand fly colony was established from specimens captured at a leishmaniasis endemic area in Madrid [18]. Salivary glands were dissected from five to seven-day-old adult female flies and stored in Tris-NaCl buffer (20 mM Tris, 150 mM NaCl, pH 7.4) in groups of 20 salivary glands in 20 μl. Salivary gland homogenate (SGH) was obtained by disrupting the glands through three repeated freezing and thawing cycles. SGH was immediately used or stored at -20°C.

### 2.2. Recombinant salivary proteins

Two rSP from *P*. *perniciosus* were used in this study. Recombinant proteins were only used in sera samples from mice. Recombinant apyrase rSP01B (GenBank ID: KF178455) and D7-related protein rSP04 (GenBank ID: KF178456) were expressed and purified under denatured conditions, and tested in their refolded form [19]. Proteins were quantified by Bradford method [20].

### 2.3. Ethical statement

BALB/c mice and New Zealand White female rabbits were housed at the animal facilities of ISCIII. Animals were treated humanely and cared for in accordance with standards specified in the Guide for Care and Use of Laboratory Animals. Experimental protocols were approved by the Bioethics and Animal Welfare Committee of Instituto de Salud Carlos III, Madrid, Spain (CBBA/4.2-PA 225/08), which complies with all relevant European Union and international guidelines for experimental animals. Animal health status was checked once a week and alleviation of the stressful/painful experimental procedure or providing analgesics was always taken under veterinarian prescription. A protocol of humane endpoint was ready in case of animals became severely ill/moribund prior to the experimental endpoint. This protocol was activated when one of the following signs were notified: weight loss of 20%, inability to eat, drink or rise, presence of a tumor larger than 10% of the animal size and severe deterioration of body condition score.

### 2.4. Exposure of mice and rabbits to sand fly bites

**Fig 1:**
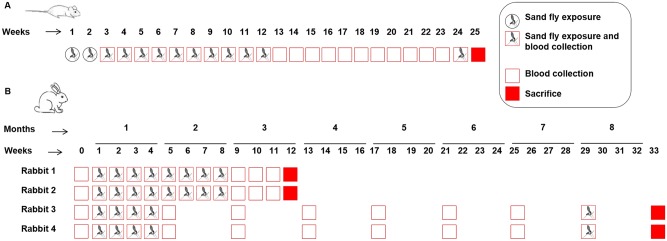
Immunization schedule. **(A)** BALB/c mice were experimentally exposed to the bites of 150 *P*. *perniciosus* during twelve weeks and followed during another twelve weeks. A new sand fly exposure was carried out at week 24 to evaluate a B cell recall. **(B)** Rabbits were exposed to 500 *P*. *perniciosus* bites for eight weeks and followed for a total period of twelve weeks (Rabbits 1 and 2) or exposed for four weeks and followed during seven more months (Rabbits 3 and 4). A new sand fly exposure was performed at week 29 and animals were bled the following week (33).

**Fig 2:**
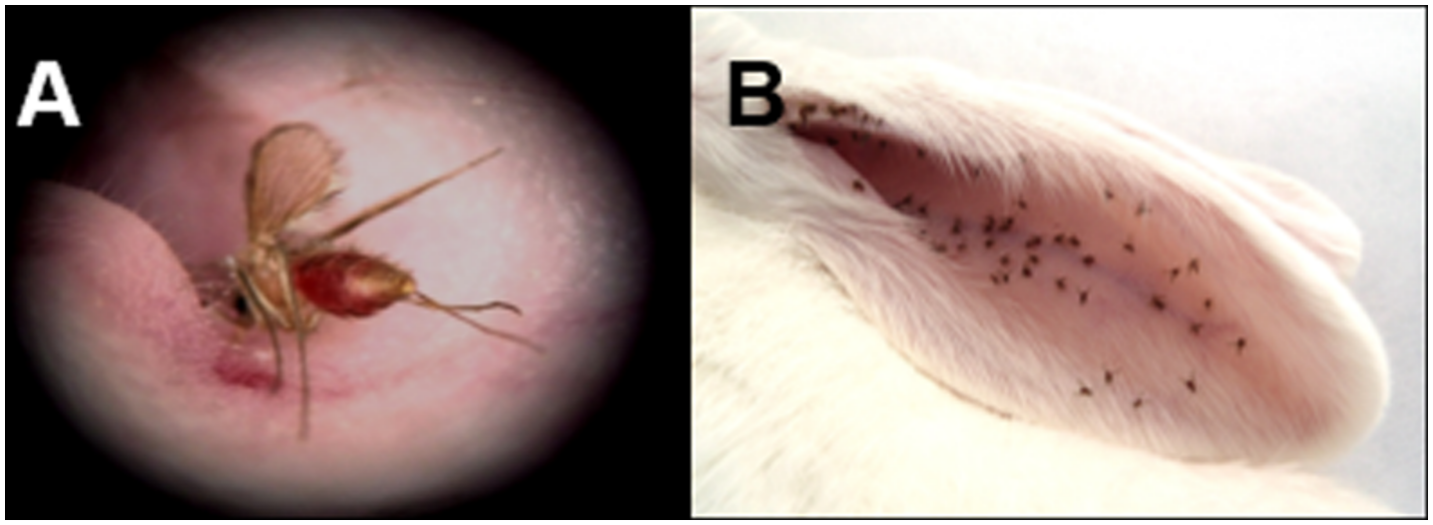
Detailed pictures of the immunization process. Female *P*. *perniciosus* feeding on the ear of anesthetized animals. (**A)** Mouse. (**B)** Rabbit.

Mice exposure model was used as a tool to compare immune responses against salivary gland homogenate and individual salivary proteins. Five-week-old-female BALB/c mice (n = 12) and New Zealand White female rabbits of 2.5 kg (n = 6) were used. Eight mice were anaesthetized (ketamine 150 mg/kg and xylazine 15 mg/kg, intraperitoneally) and individually exposed to 150 five-to-seven-day-old *P*. *perniciosus* sand fly females once a week (Fig 1A). A group of four mice were followed as negative controls. In the case of rabbits, immunization schedule was chosen in order to follow the animals for the period corresponding to the non-sand fly season in Madrid, Spain (6–7 months), after their serum had reached the maximum anti-saliva antibody levels (sand fly season). Four rabbits were sedated with a blend of ketamine 15 mg/kg and xylazine 3 mg/kg intramuscularly and individually exposed to 500 sand fly females once a week (Fig 1B). Two other rabbits served as negative controls, being bled at the same time as the exposed ones. After a non-exposure period, animals were newly exposed to sand fly bites to evaluate the presence of a B cell recall. Details of mice and rabbit immunizations are shown in Fig 2.

Ketamine and xylazine dosage were adjusted as the animal weight changed in order to accurately provide anesthesia. Before each animal was introduced into the cages containing the sand flies, we always checked that the animal was completely asleep and therefore could not feel any pain. After immunizations, animals were monitored for anesthesia recovering, and only when they were awake enough to walk they were put back to their cages. Engorged sand flies fed on each animal were counted and sand fly mortality was recorded one day after blood-feeding to evaluate whether ingestion of anti-saliva antibodies could interfere with sand fly viability. Animals were regularly bled and sera were separated from plasma by centrifugation (2,500 rpm; 20 min.; 4°C). Due to the limited amount of mice sera, only IgG antibody levels were measured with sera collected at all sampling points. Mice sera collected at odd weeks were used to analyze IgG subtypes antibody levels (IgG1 and IgG2a) and sera collected at even weeks served for Western blot (WB) analysis.

At the end of the experiment, all animals were euthanized by intramuscularly providing a mixture of ketamine and xylazine (same doses than the ones specified for anesthesia) followed by cardiac exsanguination.

### 2.5. ELISA

Specific anti-*P*. *perniciosus* saliva antibody response was measured by indirect ELISA, according to Rohoušová et al. with minor modifications [21]. Sera were diluted (1:50) in 2% skimmed milk and incubated at 37°C during 3 h. For mouse IgG subtype studies (IgG and IgG2a) sera were diluted 1:200. Peroxidase-conjugated antibodies were incubated for 1 h at 37°C (goat anti-mouse IgG 1:500, goat anti-mouse IgG1 1:10.000, goat anti-mouse IgG2a 1:1.000, goat anti-rabbit IgG 1:2.500; AbD Serotec and goat anti-rabbit IgM 1:20.000; Bethyl Laboratories). Plates were developed with orthophenylendiamine in McIlwein phosphate-citrate buffer (pH 5.5) in the presence of H_2_O_2_. The reaction was stopped with 10% H_2_SO_4_ and absorbance was measured at 492 nm using an ELISA reader (ELx800, BioTek, Winooski, VT). Each serum was tested in duplicate. The control wells were coated with SGH, but no serum was added. IgG antibody levels were reported as adjusted optical density (aOD), calculated for each serum as a mean OD value of the duplicated wells minus the OD value of the control wells. ELISA experiments were repeated at least twice.

### 2.6. Western blot

Salivary glands (20 glands per well) were separated by SDS-PAGE on 12% polyacrylamide gels under denaturing conditions using the Mini-ATTO apparatus (Biogen). Proteins were either stained by Coomassie brilliant Blue (Bio-Rad) or transferred to PVDF membranes that were cut into strips (area corresponding to one well of the 12 well-comb was cut into 4 strips) and blocked overnight at 4°C with 5% milk in Tris-Tw buffer (20 mM Tris, 150 mM NaCl, 0.1% Tween^®^ 20; pH 7.4) at 4°C. After washing in Tris-Tw, membranes were incubated for 1 h with diluted sera (mice: 1:100; rabbit: 1:50). The strips were further washed and incubated with peroxidase conjugated goat anti-mouse IgG (1:500, AbD Serotec) or goat anti-rabbit IgG (1:2.500, AbD Serotec). Immunogenic protein bands were visualized by substrate solution with diaminobenzidine and reaction was stopped with distilled water.

### 2.7. Statistical analysis

The non-parametric Mann-Whitney U test was performed for statistical analysis. The evaluation of booster effect was assessed by comparison of antibody levels before and after the last exposure to sand fly bites (weeks 23 and 25 for mice and weeks 25 and 33 for rabbits). Non-parametric Spearman rank correlation matrix was used for correlation analysis. Statistical tests were performed using Prism version 5 (GraphPad Software, Inc., San Diego, CA). The p-value was set at 0.05.

## Results

### 3.1. Kinetics of anti-saliva antibodies in BALB/c mice

**Fig 3:**
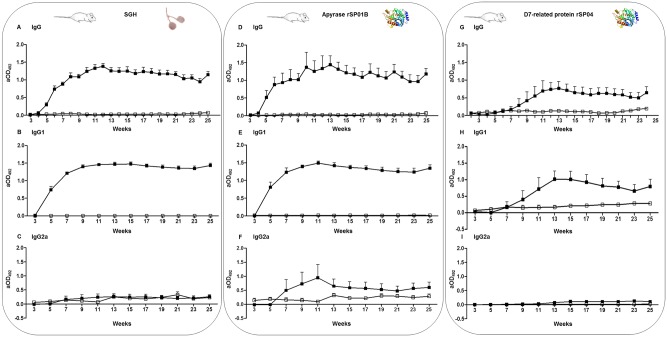
Humoral responses against *P*. *perniciosus* salivary gland homogenate and recombinant salivary proteins detected in sera of mice. **(A), (B), (C)** IgG, IgG1 and IgG2a antibody levels against *P*. *perniciosus* SGH. **(D), (E), (F)** IgG, IgG1 and IgG2a antibody levels against the recombinant apyrase rSP01B. **G, H, I)** IgG, IgG1 and IgG2a antibody levels against the recombinant D7-related protein rSP04. Open circles symbolize the non-exposed mice group while closed circles indicate the immunized mice group. Bars represent the geometric means. Antibody levels are expressed as adjusted optical density measured at 492 nm (aOD_492_).

IgG anti-saliva antibody levels detected in sera of immunized mice gradually increased during the 12 week-period of exposure to sand fly bites, reaching detectable levels from the fifth week onwards (p < 0.05). After the exposure period, IgG levels were maintained or slightly decreased. A new contact with sand fly bites after the follow-up period resulted in a significant increased level of IgG anti-saliva antibodies (p < 0.05; Fig 3A).

IgG1 antibody levels showed similar kinetics than the ones observed for total IgG, being detectable from the fifth week. Likewise, IgG1 levels were maintained during the follow-up period and slightly increased after a new exposure to sand flies (week 25, Fig 3B). However, this rise was not statistically significant (p = 0.1797). Conversely, anti-saliva IgG2a antibodies from exposed mice did not differ from IgG2a levels detected in sera of non-exposed animals (Fig 3C). No anti-saliva IgG, IgG1 or IgG2a antibodies were observed in sera of non-exposed animals.

Anti-saliva IgG and IgG1 antibody levels positively correlated with the sum of blood-fed female sand flies during the immunization phase (IgG: r = 0.9982, p < 0.0001; IgG1: r = 1, p < 0.0001). The average number of blood fed females per mouse was 943 ± 147. No relationship between the gradual increase of anti-saliva IgG antibodies and a rise in mortality was observed in sand flies repeatedly fed on the same mouse (p > 0.05).

### 3.2. Kinetics of antibodies against recombinant salivary proteins in BALB/c mice

In mice, total IgG levels against recombinant apyrase rSP01B steadily increased along the immunization process showing similar kinetics than IgG antibodies against SGH (Fig 3D), but significant detectable levels appeared as soon as at the fourth week of experiment (p < 0.05). IgG levels of anti-rSP01B correlated with IgG anti-SGH levels (r = 0.8760; p < 0.0001). However, a greater variability among anti-apyrase IgG response was observed in comparison with anti-SGH IgG. Levels of IgG1 antibodies against rSP01B also reassemble to anti-SGH antibody IgG1 levels (r = 0.8609; p < 0.0001; Fig 3E) while IgG2a showed variable values (Fig 3F). Concretely, some sera of immunized mice presented high IgG2a antibody levels, similar to IgG or IgG1 while other mice sera presented close IgG2a values to non-exposed mice along both immunization and follow-up periods.

IgG and IgG1 antibody levels against recombinant D7-protein SP04 smoothly increased along the immunization process yielding detectable antibody levels at week 9 and peaking at weeks 13–14 respectively (Fig 3G and 3H). Anti-rSP04 levels correlated with anti-SGH response (IgG: r = 0.4665, p < 0.0001; IgG1: r = 0.3577, p < 0.0001). No IgG2a response against rSP04 was detected (Fig 3I).

The relationship between antibody responses against rSP and the sum of female sand flies that fed on those animals were studied during the immunization phase as performed with anti-SGH antibody levels. Similarly, a positive significant correlation (p < 0.0005) was found between these two parameters in the case of anti-rSP01B antibody levels (IgG: r = 0.9719; IgG1: r = 1; IgG2a: r = 0.9636) and anti-rSP04 antibody levels (IgG: r = 0.9638; IgG1: r = 0.8909; IgG2a: r = 0.9636).

### 3.3. Kinetics of anti-saliva antibodies in rabbits

**Fig 4:**
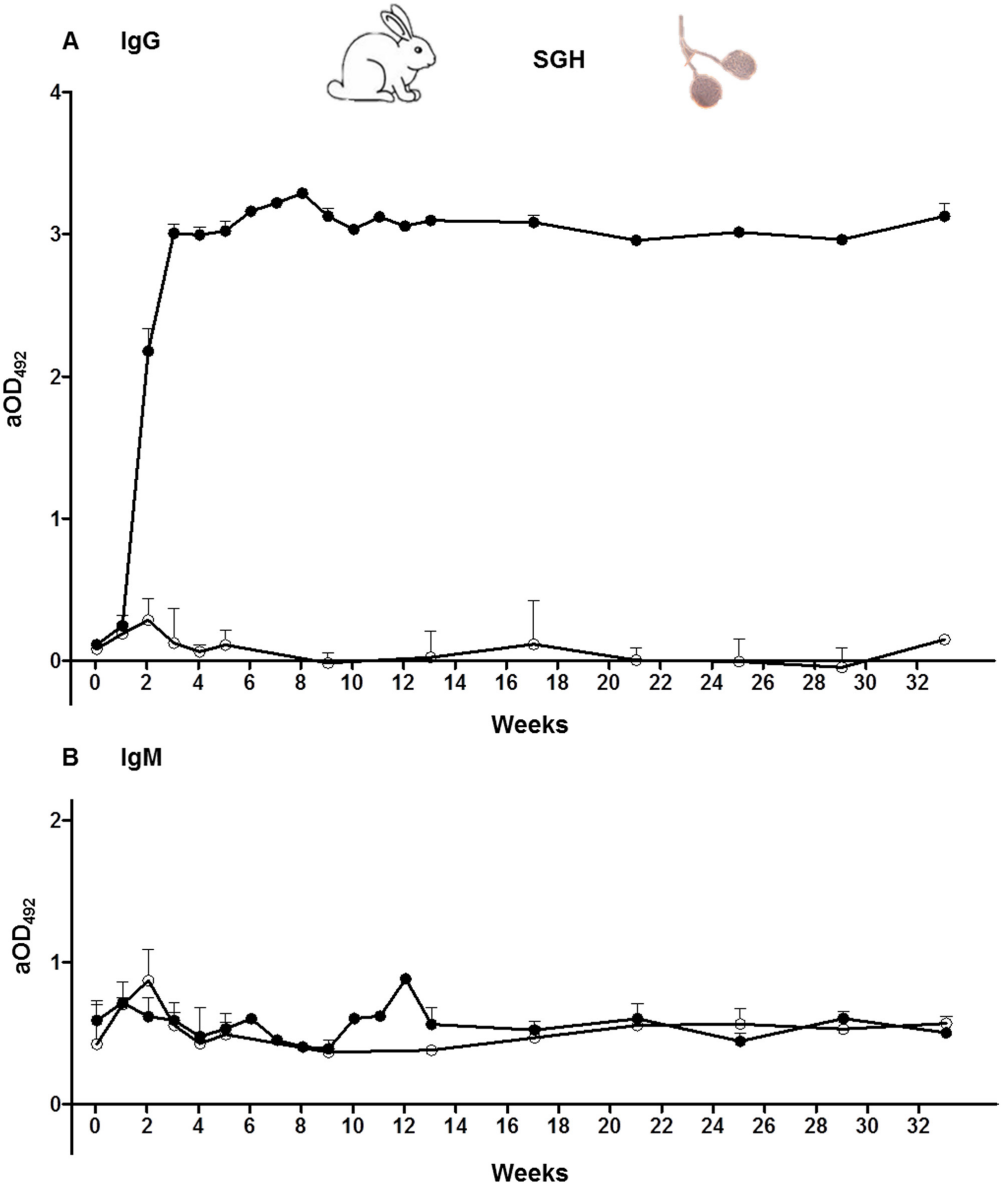
Humoral responses against salivary gland homogenate detected in sera of rabbits. **(A)** IgG antibody levels. (**B)** IgM antibody levels. Open circles symbolize the non-exposed rabbit group while closed circles indicate the immunized rabbit groups (Rabbits 1–4). Bars represent the geometric means. Antibody levels are expressed as adjusted optical density measured at 492 nm (aOD_492_).

IgG anti-saliva antibodies in the serum of rabbits were detectable from the second week of immunization, reaching high OD values (OD = 3). After this steep increase, anti-saliva IgG levels continued to rise and, thereafter, remained constant at very high values throughout the exposure phase and during the following seven months (Fig 4A). After a new contact with salivary antigens at week 29, anti-saliva IgG antibodies slightly increased. IgG levels positively correlated with the sum of blood-fed sand fly during the immunization phase (r = 0.9762; p < 0.0001). The average number of blood fed females per rabbit was 1,949 ± 920. Similar to mice sera, no relation was found between the increase of anti-saliva IgG values in sera of bitten rabbits and blood-fed sand fly mortality, recorded one day after blood-feeding (p = 0.1168).

In addition, IgM anti-saliva levels were investigated. No correlation was found between the progress of the immunization process and IgM response (Fig 4B; p = 0.1920).

### 3.4. Western blot

**Fig 5:**
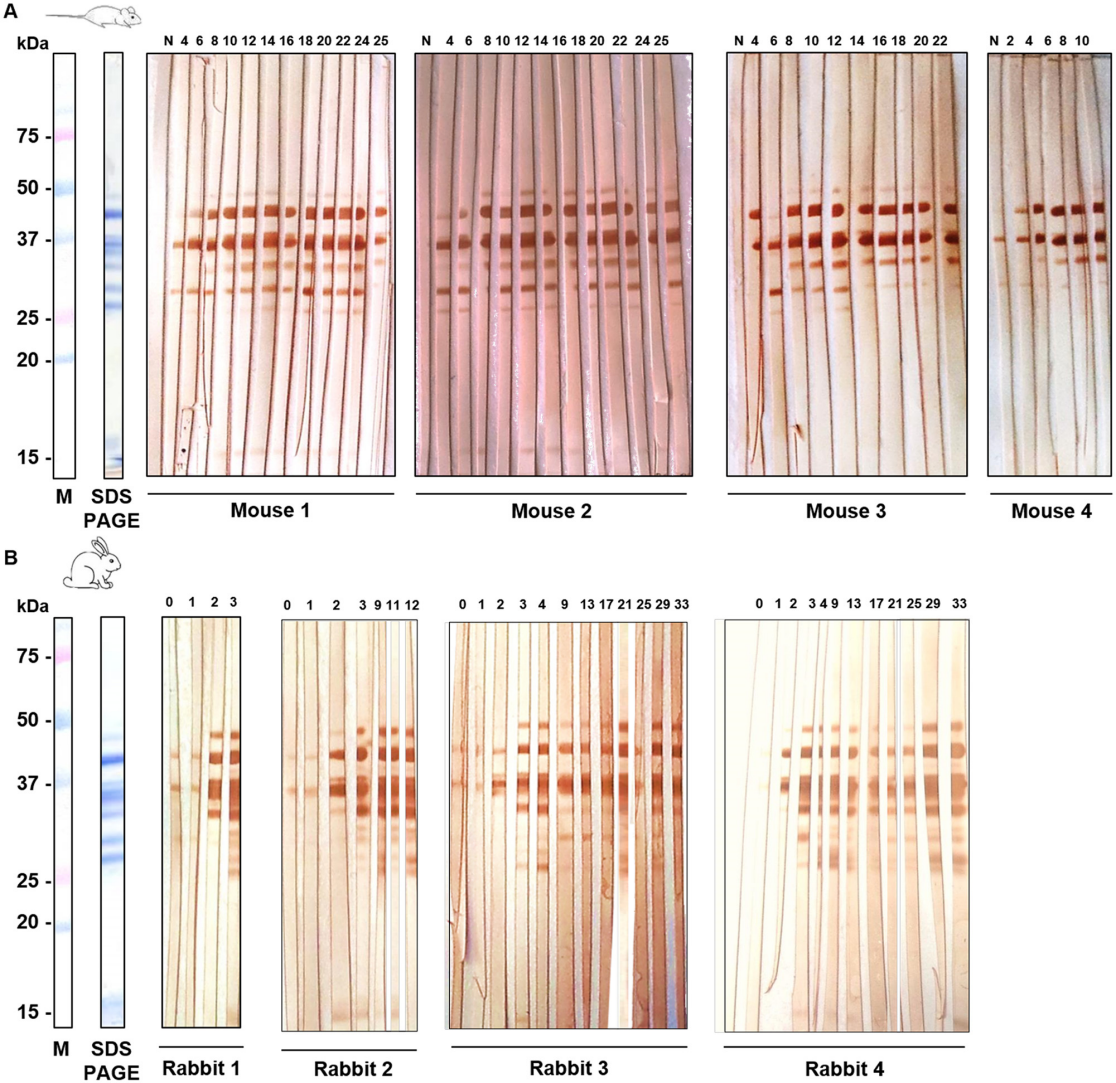
Recognition of *P*. *perniciosus* salivary proteins by representative sera of mice and rabbits by WB. Twenty salivary glands were loaded in each well, separated by SDS-PAGE and either Coomassie-stained or transferred to PVDF membranes. The area of membrane corresponding to each well was cut into 4 strips and incubated separately with mice or rabbit individual sera. **(A)** WB of *P*. *perniciosus* SGH with sera of immunized mice at weeks 4, 6, 8, 10, 12, 16, 18, 20, 22, 24 and 25. (N) Negative serum from non-exposed mice. **(B)** WB of *P*. *perniciosus* SGH with sera of immunized rabbits at weeks 0, 1, 2, 3, 4, 9, 13, 17, 21, 25, 29 and 33. (M) Molecular weight marker Precision Dual Xtra Plus (Bio-Rad).

Mice and rabbit sera showed different reactivity to *P*. *perniciosus* salivary proteins. Recognition of salivary antigens and intensity of the reaction increased along the sand fly exposure period when testing sera of both animals. The recognition signal was maintained for most of the protein bands during the follow-up period (Fig 5).

For mice, sera of animals that have received four sand fly exposures only recognized the most abundant proteins (bands at 35 and 29 kDa). From the week sixth the whole protein recognition pattern was reached in all cases (48, 44, 37, 35, 33, 29, 27 and 15 kDa) and maintained with different intensities along the follow-up period. Certain degree of variability among sera of different mice was observed for the recognition of protein bands at 29, 27 and 15 kDa. Control serum samples from non-exposed animals confirmed the specificity of the reaction (Fig 5A).

Rabbit sera recognized the same protein bands as mice´s plus other two bands at 41 and 24 kDa. At weeks 0 and 1 some faint unspecific signals were observed at the molecular weight of the most abundant proteins (44 and 35 kDa). However, specific recognition was detectable after the second sand fly exposure by all sera of immunized rabbits. 44, 37 and 15 kDa bands showed early recognition, as soon as at the second week. Conversely, other antigenic bands that were visualized at molecular weights of 41, 27 and 24 kDa appeared after the third or fourth week of exposure to sand fly bites (Fig 5B).

## Discussion

In the present work, kinetics of antibodies against *P*. *perniciosus* saliva and rSP were studied analyzing sera of experimentally bitten animals. In both BALB/c mice and rabbits, animals immunized through the bites of uninfected *P*. *perniciosus* showed a specific humoral response against saliva of this sand fly species. Rabbits are well known immune responders and normally used as animal models for polyclonal antiserum production [22], thus, developed a faster anti-saliva humoral response than mice and their sera recognized more salivary proteins. In both mice and rabbit cases, a positive relationship between the anti-saliva antibodies and the number of fed sand flies was established, highlighting the link between antibody production and the level of exposure. This association has been previously observed in mice and dogs and corroborates their use as epidemiological marker of sand fly exposure [10, 11].

Anti-saliva IgG antibodies detected in sera of mice smoothly rose until the plateau was reached which persisted for at least 24 weeks. Similar kinetics were described in BALB/c mice exposed to *Phlebotomus papatasi* bites where IgG levels were detectable from the fourth week of immunization and antibodies were maintained in sera of animals for the 24 weeks of the study [11]. Repeated exposure to *P*. *perniciosus* saliva did not elicit an IgG2a response in BALB/c mice but produced high levels of IgG1 subtype whose kinetics reassembles to IgG levels. Such a predominance of IgG1 antibodies with no IgG2a production suggests a polarization towards a Th2 immune response. Our results matched other studies in which saliva of *P*. *papatasi*, *Phlebotomus ariasi* and *Lutzomyia longipalpis* have shown to produce immediate hypersensitivity reactions mediated by the humoral Th2 response [11, 23, 24]. IgG2a, IgG2b, IgG3 antibody levels were not studied as other authors had not observed any specific production in sera of immunized BALB/c mice [11, 24]. Accordingly, detection of IgE was discarded as IgE levels seemed to be highly variable and did not correlate with the level of sand fly exposure in mice, dogs and humans [10, 11, 13, 25].

In this work, kinetic studies of antibodies against two *P*. *perniciosus* rSP were conducted with sera of BALB/c mice. We found that the two analyzed recombinant proteins elicited different immune responses. *P*. *perniciosus* SP01B belongs to the apyrase family which comprises enzymes present in the saliva of sand flies and bed bugs acting as anti-hemostatic factors [26]. Anti-apyrase rSP01B antibodies showed similar kinetics to anti-SGH antibodies, being the IgG1 subclass the predominant response with similar detectable time points and antibody levels along the process. It elicits an early immune response as anti-rSP01B antibodies are detectable one week before anti-SGH antibodies. Apyrases from different sand fly species such as *P*. *perniciosus*, *Phlebotomus sergenti*, *P*. *papatasi*, *P*. *arabicus* and *L*. *longipalpis* are recognized by sera of experimentally bitten mice or dogs [10, 11, 13, 27–33] and also by sera of naturally exposed human, dogs, hares or rabbits living in endemic areas [10, 14, 21, 28, 33]. Both *P*. *perniciosus* recombinant apyrases rSP01 and rSP01B successfully worked as sand fly exposure indicators in a pilot study with experimentally bitten mice and dogs [31]. However, in a recent longitudinal study with naturally exposed dogs from an endemic site for canine leishmaniasis in Italy, the combination of the yellow protein SP03B and the apyrase rSP01 did not show better performance than the yellow protein by itself as marker of exposure to *P*. *perniciosus* bites [15]. In this study, antibodies against whole *P*. *perniciosus* saliva, rSP03B and rSP03B + rSP01B were followed during two transmission seasons providing useful data on kinetics of anti-saliva response which corresponds to a seasonal pattern [15]. Unfortunately, these authors did not show the kinetics of canine anti-rSP01 antibodies which could have been useful to validate our data with anti-rSP01B antibodies with experimentally bitten mice since *P*. *perniciosus* apyrases SP01 (ABB00906) and SP01B (ABB00907) display similar Mw and basic pI. However, potential differences in antigenicity would not be ignored as their identity on the amino acidic sequence is not greater than 67%. On the other hand, rSP04 belongs to the D7-related proteins that are widely distributed in the saliva of hematophagous arthropods and their involvement in counteracting the coagulation cascade has been suggested [34]. The increase of IgG levels against the D7-related protein rSP04 was slower than anti-SGH levels and peaked at later points, suggesting a delayed immune response to this protein. Although immunogenicity of sand fly D7-related proteins has been highlighted with sera of several vertebrates under experimental and natural exposure to bites [10, 11, 13, 14, 19, 27, 28, 30, 33, 35]it has shown a great variability according to the vertebrate host and the immunization schedule [10, 19, 31]. Together, these findings suggest that rSP04 is not an appropriate marker of exposure to sand fly bites. Noteworthy, variability in the antibody response against rSP detected in mice sera was highlighted at an individual level. It can be observed by the greater standard deviation in comparison to anti-SGH deviation. This finding was mostly appreciated with anti-apyrase IgG2a levels since some immunized animals showed no IgG2a production while other mice, under the same immunization schedule, produced high IgG2a levels indicating a polarization towards a Th1 or mixed Th1/Th2 responses. Regarding the D7-related protein rSP04, IgG and IgG1 responses also showed individual heterogeneity. Sand fly saliva consists of a complex cocktail of molecules with highly immunogenic properties [2]. Each component can drive to opposite immune responses. For instance, other authors demonstrated that different *P*. *ariasi* salivary proteins elicited variable immune responses varying from a strong DTH response to antibody production of a combination of both [23]. Indeed, such differences in immune responses leaded to indicate that distinct *P*. *papatasi* salivary proteins can prime an anti-*Leishmania major* immune response towards protection or exacerbation of disease [36]. Anti-saliva antibody levels were maintained in sera of mice during the follow-up period and a new single exposure to *P*. *perniciosus* bites derived in an enhanced antibody response highlighting a rapid memory B cell recall. Regarding a memory response, our results matched previous observation on BALB/c mice immunized with saliva of *P*. *papatasi* and humans exposed to *P*. *argentipes* and *L*. *longipalpis* [9, 11, 37].

A prompt increase of anti-saliva IgG antibody levels in sera of rabbits led to high antibody levels which remained stable at least for seven months. As far as anti-saliva antibodies as markers of exposure are concerned, it is important to know the period of time this humoral response may persist in the serum of animals in order to provide valuable information on leishmaniasis control programs efficacy. There are few studies that evaluated the long-term kinetics of anti-saliva antibodies and these have focused on mice, dogs and humans [9–11, 13–15, 37]. To date, monitoring anti-saliva humoral response to experimentally bitten rabbits represents the first analysis of IgG kinetics in leporids. It has become an important task due to their new status as *Leishmania infantum* reservoirs in the outbreak of leishmaniasis in Madrid [16, 17]. The results indicate that the antibody response may persist from the end of the period of *P*. *perniciosus* activity in Spain (late October) until the following emergence of adults (May). These results confirmed previous observations on sylvatic hares and rabbits near the leishmaniasis focus area in the Madrid region in which high anti-saliva IgG levels were detected in sera of animals captured during winter, several months after their last exposure to sand fly saliva [21].

Anti-saliva IgG antibody levels do not seem to be an accurate marker of exposure to sand fly bites in rabbits since no decrease was observed even seven months after exposure. In addition, IgM levels showed high variability and did not permit to discriminate between the exposed and control rabbit groups either at early or late time points. Anti-saliva IgM levels had never been used as markers of exposure to sand fly bites. On the other hand, IgM detection has been described as a trustful tool for early biomarkers in the case of low-level infestation of triatomines [38].

There is no general agreement for the schedule of sand fly exposure experiments. The complexity to determine a proper schedule relies on specific properties of a given ecosystem including sand fly abundance and feeding preferences. Our mice and rabbit models are based on exposure to a large dose of salivary antigen, which could have driven to favor the development of a Th2 response. The mice model was clearly intended as a tool to compare immune responses against salivary gland homogenate and individual salivary proteins. We were able to see differences between immune responses in specific salivary components despite the potential Th2 polarization due to large antigen exposure *per se*. Noteworthy, the chosen regimen for rabbit immunization (500 sand flies weekly exposure) was established according to *P*. *perniciosus* densities in the area of the leishmaniasis outbreak in Madrid calculated from the results of entomological surveys conducted in 2012 (79.5 *P*. *perniciosus*/m^2^/night) and 2013 (73.0 *P*. *perniciosus*/m^2^/night (unpublished data). In the case of rabbits, the role of this large and long-lasting antibody levels on leishmaniasis susceptibility would be an important point to address as well as a hypothetical desensitization to salivary proteins. Furthermore, recent studies have shown that changes in sand fly exposure guidelines involve notable differences in the protective effect of saliva against progression of leishmaniasis in a mouse model [12]. Therefore, more comprehensive studies on the dynamics of anti-saliva antibodies with different immunization schedules are required.

Immunogenic recognition of salivary proteins by WB showed several differences between mice and rabbit sera. These differences may be due to the variability of immune responses elicited by distinct animal species as previously observed [21, 27, 28]. In both cases the increase of antibody levels in ELISA correlated with the visualization of a positive result by WB. Antigenic bands at 35 kDa and 29 kDa were highlighted by mice sera as antigens of an early immune response. Based on their coincident molecular weight and on previous experiments, those protein bands correspond to apyrases and Par25-like proteins respectively [11, 27, 39]. A late response in mice includes the recognition of yellow proteins (bands at 48 and 44 kDa), apyrases (band at 37 kDa) and SP15-like proteins (band at 15 kDa). Moreover, D7-related proteins (band at 27 kDa) exhibited a late recognition. In rabbits, an early response corresponds to apyrase (band at 37 kDa) as well as to other protein bands at 44 and 15 kDa (yellow protein and SP15-like protein). A late response for rabbit sera is represented by the recognition of a yellow (41 kDa) and D7-related proteins (27 and 24 kDa). WB results matched the early and late response to rSP01B and rSP04 respectively which are in accordance with antibody kinetics in ELISA.

On the other hand, no apparent relationship between the gradual increase of anti-saliva antibodies and a rise in mortality was observed in sand flies repeatedly fed on the same animal. These results are consistent with the absence of relationship found between mortality of *P*. *duboscqi* fed on immunized and not-immunized hamsters, suggesting that the amount of ingested blood does not contain enough anti-saliva antibodies to interfere with the biological processes of sand flies [40].

## Conclusions

This study shows valuable data on the kinetics of *P*. *perniciosus* salivary antibody response along the process of exposure of mice and rabbits to sand fly bites. These two animal models permitted to expand the information of anti-saliva antibody kinetics due to the variability of immune responses elicited by distinct vertebrate species.

Concretely, mice model contributed to increase the knowledge on the type of immune response *P*. *perniciosus* saliva and individual proteins elicited, highlighting differences among rSP01B, rSP04 and SGH in terms of the onset and type of the immune response generated. As saliva consists of a complex cocktail of immunomodulatory molecules that may drive to opposite immune responses it becomes essential to individually characterize the humoral and cellular immunity of the salivary proteins for their independent application as *Leishmania* vaccines or epidemiological markers of exposure.

In addition, anti-saliva antibody kinetics in sera of experimentally bitten rabbits were studied for the first time. Therefore, these results provided useful information for a better understanding of the anti-saliva antibody levels found in wild leporids that have been proved to transmit *Leishmania infantum* to *P*. *perniciosus* in the focus of human leishmaniasis in the southwestern Madrid region, Spain. Further studies on measuring anti-saliva antibody levels in population sera will be important to unravel useful information regarding sand fly exposure to humans in the focus area.
